# Long-term effects of mHealth consultation services on postpartum depressive symptoms and the mediating role of loneliness: A follow-up study of a randomized controlled trial

**DOI:** 10.1017/S0033291725102596

**Published:** 2025-12-19

**Authors:** Yuki Arakawa, Kosuke Inoue, Maho Haseda, Daisuke Nishioka, Shiho Kino, Daisuke Nishi, Hideki Hashimoto, Naoki Kondo

**Affiliations:** 1 Department of Health and Social Behavior, Graduate School of Medicine, The University of Tokyo, Tokyo, Japan; 2 Department of Social Epidemiology, Graduate School of Medicine, Kyoto University, Kyoto, Japan; 3 Department of Public Health, School of Medicine, Yokohama City University, Kanagawa, Japan; 4 Department of Social Impact Assessment and Evaluation, Graduate School of Medicine, Kyoto University, Kyoto, Japan; 5 Department of Preventive Oral Health Care Sciences, Graduate School of Medical and Dental Science, Institute of Science Tokyo, Tokyo, Japan; 6 Department of Mental Health, Graduate School of Medicine, The University of Tokyo, Tokyo, Japan

**Keywords:** health consultation services, loneliness, mHealth, mental health, mediation analysis, postpartum depression, prevention

## Abstract

**Background:**

Although the short-term preventive effects of mHealth consultation intervention on postpartum depressive symptoms have been demonstrated, the long-term effects and role of alleviating loneliness on depressive symptoms remain unclear.

**Methods:**

This follow-up study extended our previous trial, which ended at three months postpartum, by continuing observation to 12 months. Participants in the original trial were randomized to the mHealth group (*n* = 365) or the usual care group (*n* = 369). Women in the mHealth group had access to free, unlimited mHealth consultation services with healthcare professionals from enrollment through four months postpartum. The primary outcome of this study was the risk of elevated postpartum depressive symptoms at 12 months post-delivery (Edinburgh Postnatal Depression Scale score of ≥9). The mediation effect of alleviating loneliness on the primary outcome was also evaluated, using the UCLA loneliness scale at three months postpartum.

**Results:**

A total of 515 women completed the follow-up questionnaires (mHealth group, 253/365; usual care group, 262/369; 70.2% of the original participants). Compared to the usual care group, the mHealth group had a lower risk of elevated postpartum depressive symptoms at 12 months post-delivery (36/253 [14.2%] vs. 55/262 [21.0%], risk ratio: 0.68 [95% confidence interval: 0.46–0.99]). Mediation analysis showed that reducing loneliness at three months post-delivery mediated approximately 20% of the total effect of the intervention on depressive symptoms 12 months post-delivery.

**Conclusions:**

mHealth consultation services provided during the early perinatal period may help alleviate depressive symptoms at 12 months postpartum.

## Background

Perinatal depression is a critical problem faced by mothers (O’Hara & McCabe, 2013), with the global prevalence of postpartum depression estimated at 17.7% (Hahn-Holbrook, Cornwell-Hinrichs, & Anaya, 2017). Perinatal depression has been linked to attempted suicide of mothers, paternal depression, and developmental delay of their offspring (Slomian, Honvo, Emonts, Reginster, & Bruyere, 2019; Wang, Li, Qiu, & Xiao, 2021). To address these public health problems, researchers have identified potentially modifiable social factors (Zhao & Zhang, 2020) and implemented several preventive efforts (Force et al., 2019; Yasuma et al., 2020). However, women who need mental support are still not adequately treated because of barriers to healthcare access, including physical distance, lack of time, and high cost (Dennis & Chung-Lee, 2006; Fonseca, Gorayeb, & Canavarro, 2015; Kingston et al., 2015). Therefore, addressing barriers is essential for preventing perinatal depression.

Recently, mobile wireless technologies for public health—commonly referred to as ‘mHealth’ (World Health Organization, 2016)—have been increasingly implemented. For example, a large randomized controlled trial (RCT) investigated the effect of automated Internet-based cognitive behavioral therapy in preventing perinatal depression and revealed that it could be effective in women with mild psychological distress (Nishi et al., 2022). Similarly, our previous RCT revealed that providing mHealth consultation services in the early perinatal periods reduced the risk of elevated depressive symptoms at three months post-delivery to a relative risk of 0.67 (Arakawa et al., 2023). Because perinatal depression often begins within four weeks after birth but can also occur during pregnancy (Tokumitsu et al., 2020; Wisner et al., 2013), initiating mHealth interventions during pregnancy is reasonable.

Although the effectiveness of mHealth consultation services for preventing early-phase depressive symptoms has been demonstrated, its long-term effect remains unclear. Recent research indicates that some experience persistent depressive symptoms throughout the first year postpartum, some experience them only in the early postpartum period, and others only in the late postpartum periods (Kikuchi et al., 2021). Given that maternal postpartum depression is associated with adverse health outcomes in children, and that longer durations of depressive episodes exacerbate this association (Stein et al., 2014), effective preventive interventions in the late postpartum period are crucial. However, research is scant on whether mHealth interventions in the early perinatal period can have long-term effects on preventing postpartum depression. Furthermore, no research has explored how patterns of postpartum depressive symptoms over the first year after birth are affected by mHealth intervention using a randomized design. Moreover, the mechanism by which mHealth intervention prevents postpartum depressive symptoms is still unclear, despite previous studies indicating the importance of social factors, such as loneliness, on maternal mental health (Cacioppo, Hawkley, & Thisted, 2010; Dennis & Dowswell, 2013; Harrison, Moulds, & Jones, 2022).

To address these knowledge gaps, we conducted a follow-up study of our previous RCT. We hypothesized that (H1) preventive mHealth consultation during the early perinatal period may have long-term effects and influence the transition patterns of depressive symptoms during the first year after birth, and (H2) reductions in loneliness resulting from the intervention may mediate its effects on maternal mental health.

To test these hypotheses, we examined: (1) the risk of elevated depressive symptoms at 12 months postpartum, (2) group differences in the transition patterns of postpartum depressive symptoms over the first year, and (3) the mediating role of loneliness at three months postpartum in the association between the intervention and depressive symptoms at 12 months postpartum.

## Methods

### Trial design and participants of the original RCT

Our original RCT was conducted in Yokohama, an urban city and one of the most populous municipalities in Japan. Yokohama City provides conventional free face-to-face perinatal care services to all mothers in ordinary situations; however, some were restricted during the COVID-19 era. The previous study enrolled pregnant women who lived in Yokohama City from September 1, 2020, to March 7, 2021. Our inclusion criteria were (1) self-reported pregnancy (expected delivery date by October 31, 2021), (2) living in Yokohama City, and (3) able to communicate in Japanese. We recruited women who visited public ward offices to register their pregnancy status or who attended mothers’ preparation classes, regardless of trimester. We also recruited pregnant women through announcements on an official website and distributed leaflets at public facilities supporting childcare and local clinics. The details of the recruitment process have previously been described (Arakawa et al., 2023). There were no exclusion criteria since a recent review highlighted the importance of universal preventive strategies (Yasuma et al., 2020). All women who accessed our study website and agreed to participate in the study provided their informed consent with baseline data on the website. Participants were immediately randomized to either the mHealth or usual care group, using 1:1 algorithmic randomization. After group allocation, the participants were sent an online questionnaire via e-mail to collect additional baseline data. In the original trial, we followed the participants and sent an online questionnaire to evaluate their depressive symptoms up to three months post-delivery. The primary outcome of the original study was depressive symptoms at three months postpartum.

### Follow-up study design and participants

This study was a follow-up study of the original RCT. Participants who were randomized to either group in the original RCT were asked at the study’s conclusion whether they would be interested in participating in additional survey-based follow-up research. Those who said yes were included in the current follow-up study and provided a survey 12 months postpartum. We analyzed data collected via an online questionnaire with Google Forms 12 months postpartum, as well as data collected in the original RCT through a research website and online questionnaires. All participants received 1,000 JPY (approximately 7 USD) in online vouchers when they completed the questionnaire 12 months post-delivery.

### Intervention

The intervention was an mHealth consultation service offered by a private company, Kids Public, Inc. In the previous study, the City of Yokohama and Kids Public, Inc. were contracted to offer mHealth consultation services to the mHealth group, from assignment to four months post-delivery. This service was provided through the LINE platform, Japan’s most popular social media application for messaging and calling (reaching approximately 95% of women of childbearing age; Institute for Information and Communication Policy, 2021). Women in the mHealth group could freely consult with health professionals using their mobile devices, with no limit on the number of consultations. The consultants comprised obstetrician-gynecologists, pediatricians, and midwives with more than three years of experience in maternity or childcare. The doctors and midwives offered general advice and emotional support regarding pregnancy and childcare, without making diagnoses or prescriptions. Women in the mHealth group could reserve available 10-minute sessions and select their preferred consultants and communication tool (voice call, text messaging/chat, or video calls) between 6 p.m. and 10 p.m., Monday through Friday. After delivery, they could also use chat consultations with midwives without time restrictions, from 1 p.m. to 5 p.m. on Mondays, Wednesdays, and Fridays. Users were regularly sent online articles with helpful information on maternity and childcare, encouraging women to use the service if they needed help.

Women assigned to the usual care group were not offered mHealth services. Instead, they were offered access to a website developed by a research team, where they could easily obtain useful information related to maternity and childcare provided by the City of Yokohama or other institutions, such as national public hospitals. From April 28, 2022, in response to the results of the previous study, some perinatal women in Yokohama City (living in Kohoku Ward) were offered the opportunity to use the mHealth service regardless of their group assignment.

### Outcome

Our primary outcome was the risk of elevated postpartum depressive symptoms 12 months after delivery. We used the Japanese version of the Edinburgh Postnatal Depression Scale (EPDS; Cox, Holden, & Sagovsky, 1987; Okano, 1996), a 10-item structured questionnaire used to screen for perinatal depression worldwide (O’Connor, Rossom, Henninger, Groom, & Burda, 2016), to assess the primary outcome. Each item was scored from 0 to 3, with total scores ranging from 0 to 30. Higher scores on the EPDS indicate increased depressive symptoms. We defined a score of ≥9 as elevated postpartum depressive symptoms, based on the validation study (Okano, 1996). We also used EPDS scores as continuous variables. In Japan, the EPDS is also used to screen for depressive symptoms during pregnancy, with a cut-off score of ≥13 reported in another validation study (Usuda, Nishi, Okazaki, Makino, & Sano, 2017).

Based on a recent study examining the transition patterns of depressive symptoms (Kikuchi et al., 2021), we categorized participants into four patterns by their depressive symptoms at three months post-delivery in the original study and at 12 months post-delivery in the current follow-up study: (1) resilient, women with no depressive symptoms at both time points; (2) recovered, those with depressive symptoms at three months but not at 12 months; (3) late-onset, those without depressive symptoms at three months but with symptoms at 12 months; and (4) persistent, those with depressive symptoms at both three and 12 months.

### Potential mediator variable

To uncover the mechanism by which the mHealth intervention decreases postpartum depressive symptoms, we selected loneliness at three months post-delivery in the original study as a potential mediator. A Cochrane review conducted in 2013 indicated loneliness as a potential target to prevent postpartum depression (Dennis & Dowswell, 2013), and participants assigned to the mHealth group in our previous study had lower loneliness at three months post-delivery. We assessed loneliness using the Japanese version of the University of California, Los Angeles Loneliness Scale, version 3 (Arimoto & Tadaka, 2019). It is a three-item scale, with each item rated on a four-point scale: (1) *never*, (2) *rarely*, (3) *sometimes*, and (4) *always.* The total score ranges from 4 to 12, with higher scores indicating greater levels of loneliness. Loneliness was assessed at baseline and three months postpartum in the previous study, and 12 months postpartum in the current study.

### Covariates

Participants’ sociodemographic data included age, parity (primipara or multipara), marital status (married or unmarried), household number, trimester of participation (first, second, and third trimester), household income, and educational attainment level. Since no women under 20 participated, we categorized participants’ ages into 20–29, 30–34, and 35 years and over, considering their age distribution. Equivalent household income was calculated and categorized into low (<4.5 million yen), intermediate (≥4.5 and ≤ 6.5 million yen), or high (>6.5 million yen) using tertile. Educational attainment was categorized into <16 years (under university) or ≥ 16 years (university or higher). In the baseline questionnaire, participants were asked whether they had a history of mental health problems, including depression, anxiety disorder, schizophrenia, or bipolar disorder. Those who reported any of these conditions were categorized as having past mental health problems. Additionally, we included EPDS and loneliness scores at baseline as covariates.

### Statistical analysis

First, as our study participants were randomized, we compared the primary outcome according to the intention-to-treat principle. We performed Fisher’s exact tests for the primary outcome and *t*-tests for the EPDS score and the UCLA loneliness scale to compare the outcomes between the groups at 12 months after childbirth, using complete case analyses. We applied modified Poisson regression analysis for the primary outcome (Zou, 2004) and multiple linear regression analyses for the EPDS score and the UCLA loneliness scales to obtain risk ratios and risk differences and to estimate 95% confidence intervals (CIs). We also compared the primary outcome with baseline adjustment. We described mHealth use during pregnancy, the first month, and after two months postpartum, along with consultation frequency across these periods, and compared depressive symptoms at 12 months postpartum by consultation frequency. Further, we used all cases with multiple imputations for missing variables as a sensitivity analysis of the main analysis. Because the missing covariates and outcomes displayed a monotone pattern, we applied a sequence of independent univariate conditional imputation methods. Baseline variables used to impute missing baseline covariates and EPDS scores at three and 12 months post-delivery included age, parity, household size, random assignment of intervention, and calculated days between the participation and expected delivery day. We generated 10 simulated data which were combined with Rubin’s rule, obtaining the estimated treatment effect and 95% CIs.

Second, to explore how patterns of postpartum depressive symptoms up to one year differed between the two groups, we described and compared the number of participants categorized in each pattern (resilient, recovered, late-onset, and persistent) by mHealth and usual care groups. Since our previous study revealed the short-term effectiveness of the mHealth intervention, it could also influence the distribution of these depressive symptom patterns for up to one year. As a sensitivity analysis, we also ran a multinomial logistic regression model to adjust for baseline covariates.

Third, we conducted a mediation analysis to evaluate whether alleviating loneliness at three months post-delivery mediated the intervention effect on the risk of elevated depressive symptoms at 12 months post-delivery. A directed acyclic graph of our mediation model is illustrated in Supplementary Figure S1. We fitted a linear regression model for the mediator and a modified Poisson regression model for the outcome. Although our intervention was randomly assigned, there were mediator-outcome confounders between loneliness at three months post-delivery and depressive symptoms at 12 months post-delivery. Therefore, we included the covariates mentioned above in our mediation model. The EPDS and UCLA Loneliness Scale scores at participation were included as continuous variables. From this model, we estimated the total effect (from the intervention to the risk of elevated depressive symptoms at 12 months post-delivery), direct effect, and indirect effect (mediated through loneliness at three months post-delivery). We calculated the proportion mediated by dividing the coefficient of the indirect effect by that of the total effect. As a sensitivity analysis of the mediation model, we additionally included the EPDS score at three months post-delivery as a covariate, assuming that depressive symptoms preceded loneliness during this early phase. All analyses were conducted using the Stata software version 16 (StataCorp). The command *‘paramed’* was used to perform mediation analysis. The 95% CIs were obtained from 1,000 bootstrapped samples.

### Project information

A previous randomized trial was executed under a contract between the City of Yokohama, Kids Public, Inc., the University of Tokyo, and other stakeholders; however, our research team conducted this follow-up study independently. The researchers were solely responsible for questionnaire construction, outcome data collection, analyses, and manuscript preparation. The study protocol was approved by the ethical review board of the University of Tokyo (No. 2019347NI).

## Results

**Figure 1.**
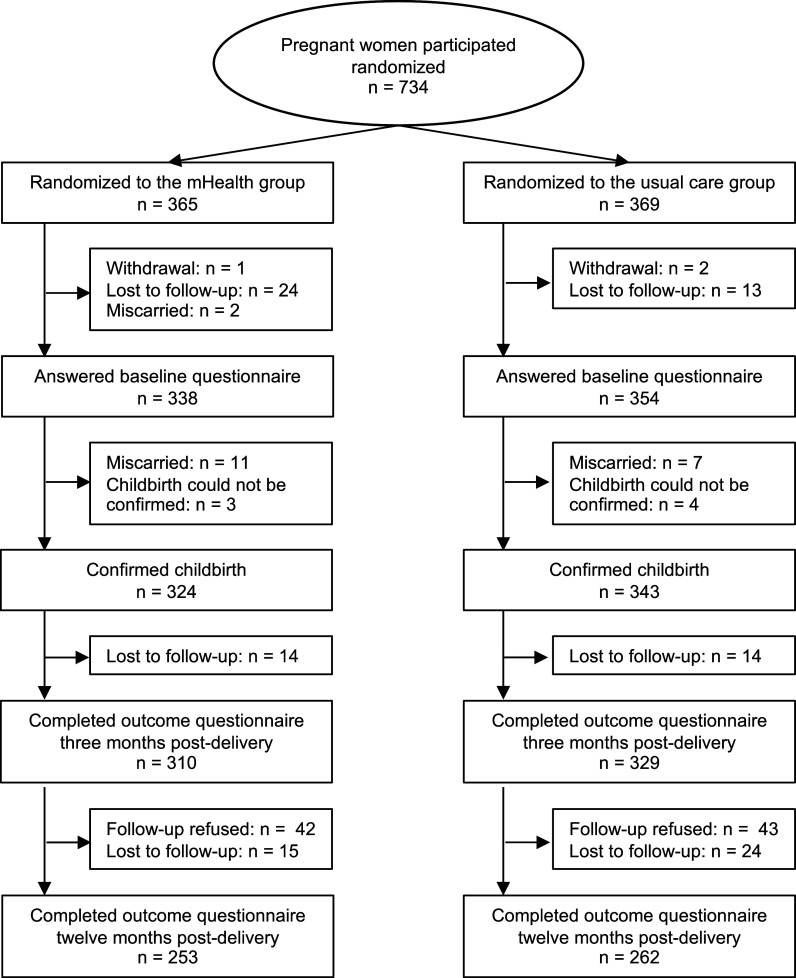
Flow diagram of the study participants.

Among 734 women allocated to the mHealth (*n* = 365) or usual care (*n* = 369) groups in a previous study, 310 and 329 women completed the three months post-delivery questionnaires in the mHealth and usual care groups, respectively. Of these, 42 and 43 women refused to participate in the current study, respectively. Therefore, we followed 268 and 286 women in the mHealth group and usual care group (554 women; 75.5% of the original participants) up to 12 months post-delivery, with 15 and 24 lost to follow-up, respectively. Consequently, 253 women in the mHealth group and 262 women in the usual care group completed the outcome assessment 12 months after delivery in the current study (515 women; 70.2% of the original participants, Figure 1). Baseline characteristics of participants who completed all questionnaires were similar between the mHealth and usual care groups (Table 1). At baseline, 5.5% of the mHealth group and 9.2% of the usual care group exhibited elevated depressive symptoms, as indicated by the EPDS score of ≥13. Loneliness levels were also comparable between the two groups at baseline.

**Table 1.** Baseline characteristics of the study participants who completed the 12-month post-delivery questionnaire (*n* = 515)

Characteristics	mHealth group (*n* = 253)		Usual care group (*n* = 262)	
Maternal age, years				
Distribution – no. (%)				
20–29	52	(20.6)	54	(20.6)
30–34	111	(43.9)	116	(44.3)
35–45	90	(35.6)	92	(35.1)
Mean [SD]	33.0	[4.1]	33.1	[4.3]
Married or partnered – no. (%)	253	(100)	262	(100)
Primipara – no. (%)	160	(63.2)	156	(59.5)
Gestational age at participation – no. (%)				
First trimester	107	(42.3)	112	(42.7)
Second trimester	40	(15.8)	54	(20.6)
Third trimester	106	(41.9)	96	(36.6)
Household number – no. (%)				
1	1	(0.4)	1	(0.4)
2	154	(60.9)	149	(56.9)
≥ 3	98	(38.7)	112	(42.7)
Equivalent household income – no. (%)				
Low (≤ 4.5 million yen)	88	(34.8)	88	(33.6)
Intermediate (> 4.5& < 6.5 million yen)	112	(44.3)	99	(37.8)
High (≥ 6.5 million yen)	52	(20.6)	74	(28.2)
Unknown	1	(0.4)	1	(0.4)
Education – no. (%)				
<16 years (under university)	60	(23.7)	52	(19.8)
≥16 years (university or higher)	193	(76.3)	210	(80.2)
Elevated depressive symptoms at baseline – no. (%)				
EPDS ≥13	14	(5.5)	24	(9.2)
Having mental health problem history - no. (%)	18	(7.1)	30	(11.5)
Loneliness, mean [SD]				
At the participation	6.8	[2.1]	6.9	[2.3]
Three months after delivery	6.8	[2.2]	7.2	[2.3]

*Note*: EPDS, Edinburgh Postnatal Depression Scale; SD, standard deviation.

Compared to women in the usual care group, women in the mHealth group had a lower risk of experiencing elevated postpartum depressive symptoms at 12 months post-delivery (36 of 253 women [14.2%] vs. 55 of 262 women [21.0%]; relative risk, 0.68 [95% CI, 0.46, 0.99]; Table 2). The mean EPDS score at 12 months post-delivery was also lower in the mHealth group than in the usual care group (4.7 vs. 5.6, mean difference: -0.83 [95% CI, −1.60 to −0.07]). We found no difference in loneliness levels at 12 months post-delivery between the two groups (7.1 vs. 7.2, mean difference: -0.19 [95% CI, −0.61 to +0.24]). When adjusting for baseline covariates, the results were qualitatively consistent with the main analysis, although the 95% CI included the null (relative risk, 0.72 [95% CI, 0.49, 1.05]). About half of the women used mHealth during pregnancy, in the first month postpartum, and after two months postpartum, with most having zero to two consultations (Supplementary Table S1, Figure S2). No differences in depressive symptoms at 12 months postpartum were observed across consultation frequency categories (Supplementary Table S2). Our sensitivity analysis of the main analysis, imputing all missing variables, also showed a similar direction of the intervention effect with the main analysis; however, the 95% CIs of the sensitivity analysis included null (relative risk, 0.71 [95% CI, 0.51, 1.00]).

**Table 2.** Postpartum depressive symptoms, score of EPDS, and loneliness at 12 months post-delivery between the mHealth and usual care group

Outcome, no. (%) or mean [SD]	mHealth group (*n* = 253)	Usual care group (*n* = 262)	Relative risk^a^(95% CI)	Mean difference^b^(95% CI)	*P* value
Elevated postpartum depressive symptoms (EPDS ≥9)	36/253 (14.2)	55/262 (21.0)	0.68 (0.46, 0.99)	–	.05
Scores of EPDS	4.7 [4.0]	5.6 [4.7]	–	–0.83 (−1.6, −0.07)	.03
Loneliness	7.1 [2.3]	7.2 [2.6]	–	–0.19 (−0.61, 0.24)	.38

*Note*: CI, confidence interval; EPDS, Edinburgh Postnatal Depression Scale; SD, standard deviation.

a Relative risks were calculated using modified Poisson regression models.

b Mean differences were calculated using linear regression models.

From the assessment of the transition patterns of depressive symptoms, of the 253 women in the mHealth group and 262 in the usual care group, 194 (76.7%) and 173 (66.0%) were classified as resilient, 23 (9.1%) and 34 (13.0%) as recovered, 23 (9.1%) and 25 (9.5%) as late-onset, and 13 (5.1%) and 30 (11.5%) as persistent, respectively (Table 3). Compared to the women in the usual care group, more women in the mHealth group were classified as resilient in the postpartum period. The proportions of women categorized in the recovered and persistent pattern were lower in the mHealth group, and the difference was evident in the persistent one. In contrast, we found no apparent difference in the proportion categorized in the late-onset depressive symptom pattern between the two groups. When adjusted for baseline covariates, the proportion categorized as the persistent pattern was lower in the mHealth group than in the usual care group (Supplementary Table S3).

**Table 3.** Differences in the transition patterns of postpartum depressive symptoms between the mHealth and usual care groups

Patterns	Postpartum depressive symptoms^a^		mHealth group(*N* = 253)	Usual care Group (*N* = 262)
	At 3 months	At 12 months	n (%)	n (%)
Resilient	−	−	194 (76.7)	173 (66.0)
Recovered	+	−	23 (9.1)	34 (13.0)
Late-onset	−	+	23 (9.1)	25 (9.5)
Persistent	+	+	13 (5.1)	30 (11.5)

a Postpartum depressive symptoms were defined as present with the Edinburgh Postnatal Depression Scale scored nine or higher.

In the mediation analysis, the total effect was estimated to be 0.70 [95% CI, 0.45, 1.08] and the indirect effect (mediated through loneliness at three months post-delivery) was 0.93 [95% CI, 0.81. 0.99] (Table 4). The proportion mediated by reducing loneliness was estimated to be 19.8%. Our sensitivity analysis of the mediation model with adjustment for the EPDS score at three months post-delivery showed consistent direction; however, the proportion mediated was approximately halved (total effect, 0.75 [95% CI, 0.44, 1.16]; indirect effect, 0.97 [95% CI, 0.87, 1.01]; proportion mediated, 10.9%).

**Table 4.** Direct and indirect (through loneliness) effects of mHealth consultation services on the risk of elevated postpartum depressive symptoms (EPDS ≥9)

Elevated postpartum depressive symptoms (EPDS ≥9)	Total effect		Direct effect		Indirect effect		% Mediated^a^
	RR	95% CI	RR	95% CI	RR	95% CI	
Intervention	0.70	0.45, 1.08	0.75	0.49, 1.15	0.93	0.81, 0.99	19.8

*Note*: CI, confidence interval; EPDS, Edinburgh Postnatal Depression Scale. The outcome model was modified Poisson regression with baseline adjustment. The mediation model was a linear regression with baseline adjustment. The adjustment variables were age, parity, trimester, household number, income, education, depressive symptoms at participation, loneliness at participation, and history of mental health problems.

a We calculated the proportion of the total effect that was mediated through loneliness at three months post-delivery by dividing the coefficient of indirect effect by that of total effect.

## Discussion

Compared to the usual care group, the mHealth group had a lower risk of elevated postpartum depressive symptoms by one-third at 12 months post-delivery, despite our intervention being provided only from participation to four months post-delivery. Although the original study was randomized, the mHealth group had fewer participants with depressive symptoms at baseline in this follow-up due to attrition. However, the results were qualitatively consistent after adjusting for baseline covariates. When exploring differences in the transition patterns of depressive symptoms during the first year postpartum between the two groups, we found that the proportions of the recovered and persistent patterns were lower in the mHealth group. Our mediation analysis estimated that the effect of mHealth consultation services on the risk of elevated depressive symptoms at 12 months post-delivery was 10–20% mediated by reducing loneliness at three months post-delivery.

Our follow-up study suggests that early perinatal mHealth intervention may help alleviate postpartum depressive symptoms during the first year after delivery. Studies indicated some women suffer from their depressive symptoms in the late phase of postpartum (Chow et al., 2019; Fisher et al., 2019; Putnick et al., 2020), requiring effective interventions to reduce these burdens; however, most of the RCTs of perinatal mHealth interventions did not explore their impact on late-phase symptoms. For example, a recent review of digital interventions for preventing postpartum depression included 14 studies; of these, only two assessed outcomes at six months postpartum, and only one assessed outcomes at one year postpartum (Lewkowitz et al., 2024). Similarly, a study reviewing app-based interventions included 16 studies, of which only one assessed the outcomes at one year postpartum (Miura et al., 2023). In addition, the two studies that assessed symptoms at one year found no clear effects, suggesting limited evidence for the long-term effectiveness of mHealth interventions. Our study suggests that early perinatal mHealth support may help prevent the worsening of depressive symptoms in the later postpartum period. Several mechanisms may explain the long-term effects of early perinatal mHealth consultation services on depressive symptoms. Women in the mHealth group may have obtained general skills and knowledge about childcare, such as breastfeeding and child development, as well as individualized informational and emotional support, which could help them deal with issues in the later postpartum periods. From the perspective of the transactional model of stress and coping (Folkman, Lazarus, Gruen, & DeLongis, 1986), overcoming problems in the early perinatal period with professional support can enhance coping abilities in managing childcare issues. This, in turn, may contribute to preventing depressive symptom elevation during later postpartum periods. Help-seeking experience is another factor that may explain our findings. Several studies have emphasized that perinatal women do not know that their feelings are not normal or how to deal with them (Goodman, 2009; Kingston et al., 2015). mHealth consultation services could reduce access barriers to seeking help and consultation on their feelings, which would lead to their recognition that it is better to seek help early. During the COVID-19 era, new private and public consultation services emerged, including one launched by Yokohama City in the later phase of this follow-up study. Women in the mHealth group may have increased their help-seeking behaviors through these services, based on positive early perinatal experiences. These mechanisms could have been achieved by the features of the mHealth consultation service, especially its timeliness and appropriateness through individualized support (Sakamoto et al., 2022).

Our exploratory analysis suggested that the mHealth service may help alleviate early-phase and persistent depressive symptoms. A previous Japanese study reported that some women experience depressive symptoms in the early postpartum period and recover naturally (recovered pattern; Kikuchi et al., 2021). The lower proportion of this pattern in our study suggests that the mHealth intervention may have prevented an elevation in depressive symptoms during the early perinatal period and maintained lower levels over one year, thereby reducing the number of women who would otherwise have been categorized as recovered without mHealth. The study also identified predictive factors for persistent depressive symptom patterns, such as higher psychological distress in early pregnancy. Our intervention, providing informational and emotional support during pregnancy, may have reduced psychological distress and, in turn, prevented persistent depressive symptoms. As a result, the mHealth intervention may have shifted women from the recovered and persistent patterns to the resilient ones. Early-phase and persistent depressive symptoms can affect offspring development (Tainaka et al., 2022). Delivering mHealth services in the early perinatal period may benefit both perinatal women and their offspring by minimizing prolonged exposure to maternal depressive symptoms throughout postpartum, which, in turn, may have a social impact from the life course perspective. In contrast, we did not find clear group differences in the late-onset pattern. Women may face challenges unique to the later postpartum period, such as child mobility or exhaustion after returning to work (Chow et al., 2019). Further studies are needed to clarify specific factors related to late-onset depressive symptoms.

Our mediation analysis suggests that alleviating loneliness can be important for preventing perinatal depression. Several studies revealed that loneliness can predict depression (Adlington et al., 2023; Kruse, Williams, & Seng, 2014). A review of preventive interventions for postpartum depression suggested the importance of reducing loneliness (Dennis & Chung-Lee, 2006), however, whether alleviating loneliness contributes to preventing postpartum depression was not empirically proven by an RCT. Our finding of the mediating effect of loneliness indicates that constructing a meaningful social connection may be important in preventing postpartum depression. Our intervention facilitated relationships and interactions with healthcare providers through mHealth, which could not be achieved through a completely automated program. Some studies indicate that human interaction in mHealth is beneficial in improving retention rates and outcomes (Mohr, Cuijpers, & Lehman, 2011; Silang et al., 2022; Torous, Lipschitz, Ng, & Firth, 2020), which may be partly explained by a feeling of connectedness and reduced loneliness (Sakamoto et al., 2022). However, the mediated proportion was less than 20%, suggesting that other factors may play a substantial role in producing the preventative effect. Changes in coping style—such as an increase in help-seeking behavior—are considered potential mediators, as such changes, once established, are likely to persist over time. We must continue seeking other factors that contribute to preventing depressive symptoms to achieve effective and replicable mHealth interventions.

This study has several limitations. First, the attrition rate of 30% may introduce bias to the results. Some participants with depressive symptoms during the postpartum period might refuse to be followed up or drop out because they could not use the mHealth consultation services in the follow-up periods, which might yield overestimation. Despite the RCT design, baseline differences in depressive symptoms due to attrition should also be considered when interpreting the findings. Second, due to the lack of time-ordering between loneliness and depressive symptoms at three months, we cannot rule out the possibility that depressive symptoms at three months confounded the mediator-outcome association. However, we found similar results in our sensitivity analysis of mediation adjusting for EPDS score at three months post-delivery, indicating that alleviating loneliness played, to some extent, a necessary role in the effect of mHealth on long-term postpartum depression. Third, some unmeasured confounders between the mediator and outcome, such as children’s temperament or work environment, may yield confounding bias. Fourth, although the EPDS is the most reliable patient-reported screening measure for postpartum depression and a well-validated scale for diagnostic interviews for perinatal depression worldwide (Levis et al., 2020; Sultan et al., 2022), we did not utilize a formal clinical diagnosis of postpartum depression as the primary outcome. Fifth, the consultation service launched by Yokohama City in the later phase of this follow-up study may have influenced depressive symptoms, as women in the usual care group also had access, though for a limited duration and proportion. Sixth, we included only women who could communicate in Japanese, so our findings cannot be extended to those with language barriers in Japan. Finally, all study participants had partners and most had high incomes and levels of education. In addition, this study was conducted during and after the COVID-19 pandemic, when face-to-face contact was partially restricted. These circumstances may limit the generalizability of our findings.

## Conclusions

In this follow-up study of RCT, we found that mHealth services may have a long-term effect in alleviating elevated postpartum depressive symptoms one year after delivery. Our findings suggest that providing mHealth consultation services focused on the early perinatal period may positively impact perinatal women’s mental health related to their offspring’s developmental delay. Interventions aimed at alleviating loneliness may contribute to the development of more effective mHealth strategies for preventing postpartum depression. Incorporating our findings into future mHealth prevention programs and evaluating their broader public health impact are warranted.

## Supporting information

Arakawa et al. supplementary material Arakawa et al. supplementary material

## Supplementary material

The supplementary material for this article can be found at http://doi.org/10.1017/S0033291725102596.
