# Supplementary material for: Long-term effects of mHealth consultation services on postpartum depressive symptoms and the mediating role of loneliness: A follow-up study of a randomized controlled trial
Source: Psychol Med. 2025 Dec 19;55:e379. doi: 10.1017/S0033291725102596 (PMC13058622; doi:10.1017/S0033291725102596)
Supplement: Arakawa et al. supplementary material [file S0033291725102596sup001.docx]

Supplementary Materials

**Table S1.** Proportion of women who used mHealth service during the early perinatal period among the mHealth group (n=253).

**Table S2.** Risk of elevated postpartum depressive symptoms (EPDS ≥9) at 12 months postpartum by total number of mHealth uses during pregnancy through three months postpartum (n=515).

**Table S3.** Differences in the transition patterns of postpartum depressive symptoms between the mHealth and usual care groups with baseline adjustment.

**Figure S1.** Causal diagram of mediation analysis

**Figure S2-A.** Distribution of the number of mHealth consultations during pregnancy among women in the intervention group (n=253).

**Figure S2-B.** Distribution of the number of mHealth consultations in the first month postpartum among women in the intervention group (n=253).

**Figure S2-C.** Distribution of the number of consultations after 2 months postpartum among women in the intervention group (n=253).

**Table S1.** Proportion of women who used mHealth service during the early perinatal period among the mHealth group. (n=253)

| mHealth use | During pregnancy | In the first month postpartum | After two months postpartum |
| --- | --- | --- | --- |
| Yes | 105 (41.5) | 103 (40.7) | 134 (53.0) |
| No | 148 (58.5) | 150 (59.3) | 119 (47.0) |

**Table S2.** Risk of elevated postpartum depressive symptoms (EPDS ≥ 9) at 12 months postpartum by total number of mHealth uses during pregnancy through three months postpartum. (n=515)

| Total number of mHealth use during pregnancy through three months postpartum ^b^ | Women with elevated depressive symptoms at 12 months postpartum, n (%) | Relative risk ^a^  (95% CI) | P value |
| --- | --- | --- | --- |
| 0 | 65/326 (20.0) | - | - |
| 1 | 7/54 (13.0) | 0.76 (0.38–1.49) | .43 |
| 2–4 | 10/77 (13.0) | 0.77 (0.43–0.35) | .35 |
| ≥ 5 | 9/58 (15.5) | 0.62 (0.31–1.21) | .16 |

Abbreviation: EPDS, Edinburgh Postnatal Depression Scale; CI, confidence interval.

^a^ Modified Poisson regression analysis with baseline adjustment was applied to calculate relative risks. The adjustment variables were age, parity, trimester, household number, income, education, depressive symptoms at participation, loneliness at participation, and history of mental health problems.

^b^ The explanatory variable was the total number of consultations, with no mHealth use as the reference category.

**Table S3.** Differences in the transition patterns of postpartum depressive symptoms between the mHealth and usual care groups with baseline adjustment.

| Patterns | Postpartum depressive symptoms | | mHealth group  (n=253) | Usual care Group (n=262) | Relative risk ratio ^a^  (95% CI) | P value |
| --- | --- | --- | --- | --- | --- | --- |
|  | at 3 months | at 12 months | no. (%) | no. (%) |  |  |
| Resilient | - | - | 194 (76.7) | 173 (66.0) | ref | - |
| Recovered | + | - | 23 (9.1) | 34 (13.0) | 0.61 (0.33, 1.15) | .13 |
| Late-onset | - | + | 23 (9.1) | 25 (9.5) | 0.74 (0.39, 1.42) | .37 |
| Persistent | + | + | 13 (5.1) | 30 (11.5) | 0.32 (0.14, 0.73) | .006 |

Abbreviation: CI, confidence interval.

^a^ Relative risk ratios and 95% confidence intervals were calculated using multinomial logistic regression models. The adjustment variables were age, parity, trimester, household number, income, education, depressive symptoms at participation, loneliness at participation, and history of mental health problems.

Ref. https://stats.oarc.ucla.edu/stata/output/multinomial-logistic-regression

**Figure S1.** Causal diagram of mediation analysis.

**
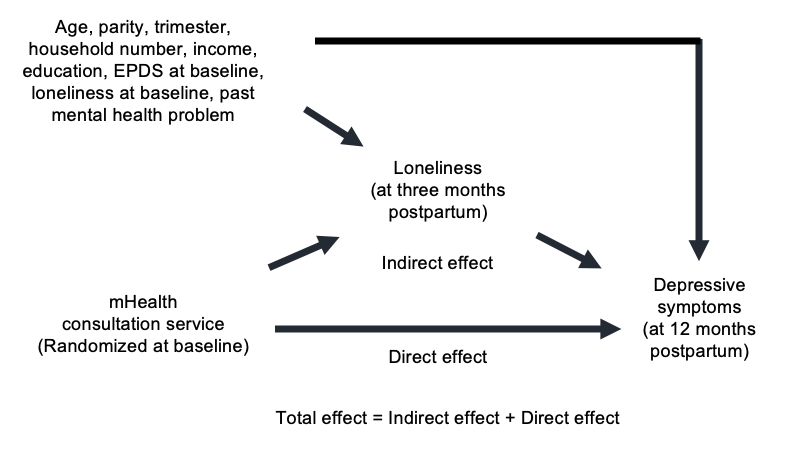
**

**
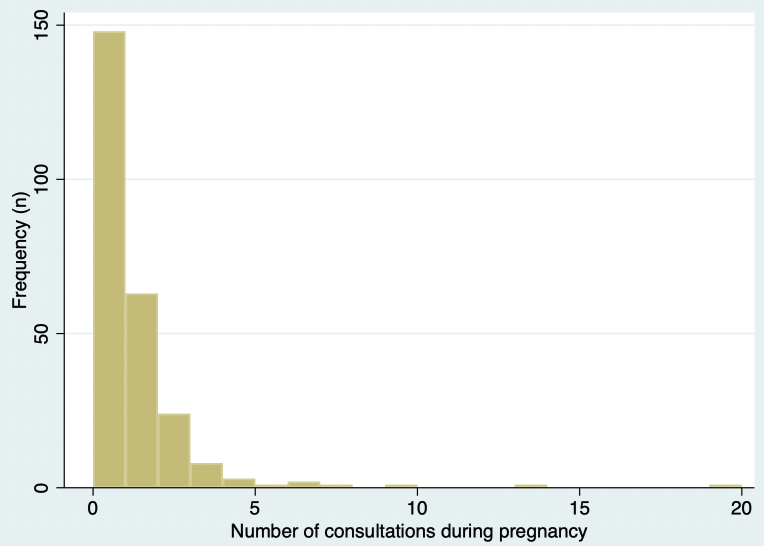
Figure S2-A.** Distribution of the number of mHealth consultations during pregnancy among women in the mHealth group (n=253).

**
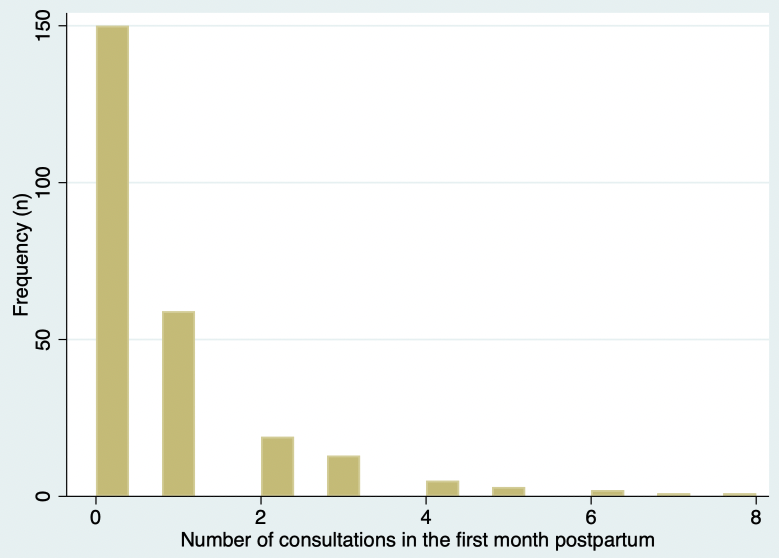
Figure S2-B.** Distribution of the number of mHealth consultations in the first month postpartum among women in the mHealth group (n=253).

**
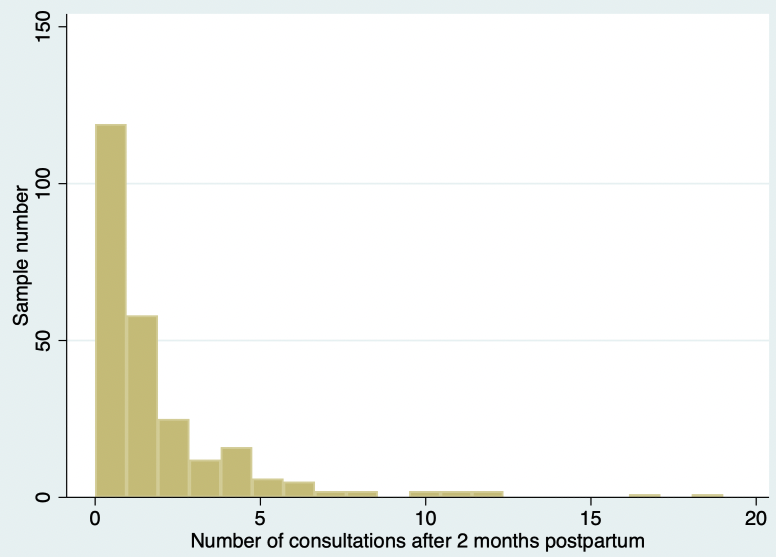
Figure S2-C.** Distribution of the number of mHealth consultations after 2 months postpartum among women in the mHealth group (n=253).
